# Advanced Sensing and Control Technologies for Autonomous Robots

**DOI:** 10.3390/s24175478

**Published:** 2024-08-23

**Authors:** Yuanlong Xie, Shuting Wang, Shiqi Zheng, Zhaozheng Hu

**Affiliations:** 1 School of Mechanical Science and Engineering, Huazhong University of Science and Technology, Wuhan 430074, China; wangst@hust.edu.cn; 2 School of Automation, China University of Geosciences, Wuhan 430074, China; zhengshiqi@cug.edu.cn; 3 ITS Research Center, Wuhan University of Technology, Wuhan 430063, China; zzhu@whut.edu.cn

## 1. Introduction

The development of advanced sensing and control technologies provides increased intelligence and autonomy for robots and enhances the robots’ agility, maneuverability, and efficiency, which has attracted growing attention in various industries and domains [1,2,3], including manufacturing [4], logistics [5], and warehousing [6].

Information about the environmental and working conditions is crucial for the safe navigation of autonomous robots [7]. Sensors play a vital role in providing data for navigation, such as map construction [8], self-localization [9], and obstacle detection [10]. The advanced sensing technologies, benefiting from the development of sophisticated sensors and innovative perception algorithms, has enabled autonomous robots to perceive accurate information with faster speed and higher precision [11,12]. This has facilitated the utilization of robots in complex scenarios [13]. A well-designed control algorithm is essential for the precision and stable navigation of an autonomous robot system. Control technology encompasses system modeling [14], parameter identification [15], tracking control [16], and cooperative control [17]. As advanced sensors and control technologies develop, autonomous robots complete tasks individually or in coordinated swarms, offering unprecedented flexibility across diverse fields of application [18,19,20]. These applications have expanded beyond traditional manufacturing and automation settings.

In the Special Issue titled “Advanced Sensing and Control Technologies for Autonomous Robots”, the latest research in cutting-edge fields and emerging topics is presented. This Special Issue invited diverse research contributions from scholars engaged in related fields, including design, modeling, and parameter identification of novel robotic systems; advanced perception, self-localization, and map-building technologies based on neural networks; intelligent path planning and formation control methods for multi-agent systems. After a rigorous review, fourteen papers, including thirteen articles and one review, were selected. They offer original ideas and feasible solutions to address critical issues in the field of autonomous robots and explore the application of the systems in smart manufacturing, autonomous driving, housekeeping, etc. Each article contributes to the advancement of specific areas, and we recommend that readers explore these articles in detail to gain a comprehensive understanding. It is our hope that this Special Issue contributes to the continued development of the robotics field.

## 2. Overview of the Published Articles

The first contribution presents a new approach to controller design for nonlinear cascade systems. These systems pose a challenge in engineering applications due to their complex dynamics and inherent uncertainty. To address this, the authors estimate the nonlinear characteristics of each subsystem by separating the steady and alternating components, using local tracking errors for model-free adaptive control. Additionally, they employ a square error correction procedure to approximate the weight coefficients and mitigate the uncertainty. This straightforward method can be readily implemented in engineering practice, providing a practical alternative to existing schemes.

In Contribution 2, Hongchao Zhang and co-authors present an advanced method for estimating the peak adhesion coefficient on unstructured pavements, which is typically challenging due to uneven surfaces and steep slopes. The authors propose an approach based on the extended Kalman filter, improving the accuracy of identifying road adhesion coefficients by incorporating an equivalent suspension model. This model optimizes the vertical wheel load calculations and adjusts the vehicle acceleration using posture data. Through multi-condition simulations with CarSim, the method demonstrates an accuracy improvement of at least 3.6%, verifying the precision and effectiveness of the designed algorithm. This robust and efficient method proves particularly beneficial for autonomous driving scenarios, enabling accurate ground adhesion data collection and sharing. It showcases the method’s practical applicability and significant potential for real-world implementation, making it a valuable contribution to the field of mobile robotics and autonomous driving.

This Special Issue presents a multifaceted exploration of SLAM technology, the foundational technology in robotics. Among the articles, Contribution 3 proposes a solution to SLAM’s difficulty in maintaining high localization accuracy over time in complex scenarios. Inspired by human neuroscience, the authors introduce NeoSLAM, a new scheme that incorporates a computational model of the brain into the traditional SLAM framework. To enhance the system’s robustness to environmental disturbances, the authors employ a hierarchical temporal memory model, optimizing the real-time performance. This innovative approach improves the adaptability of SLAM systems to dynamic environments.

A novel model for swarm behavior control is introduced through artificial empathy in Contribution 4. The authors emphasize the importance of incorporating empathy into swarm control for optimized performance and the learning mechanisms within the swarm. The research aims to validate the artificial empathy model through simulations and further development, with the goal of accurately recreating human empathy. By utilizing fuzzy set theory and similarity measures, the model emulates human empathetic decision processes in swarm control. It focuses on knowledge representation, decision-making processes, and empathetic communication, providing a comprehensive and effective framework compared to existing models. The research also highlights the significance of an open-source physical-based experimentation platform for evaluating various models and scenarios in robotics. The work lays the foundation for further exploration and development of empathetic swarm control models in diverse environments.

Contribution 5, by Da Jiang and co-authors, addresses the problem of integrated open space risk in architectural scenarios of unmanned vehicles. The authors propose an adaptive dynamic windowing approach that includes a specially designed multi-objective speed sampling window and a layered decision-making mechanism. This method enables obstacle avoidance in multiple driving modes, with speed planning commands constrained within a reasonable range. The core concept of the method is the introduction of an adaptive prediction horizon and a critical rollover speed window, which dynamically adjusts the window in high-risk environments to ensure planning safety. This approach enhances safety and effectively avoids the risks of instability associated with fast steering maneuvers.

Yongchao Zhang and his colleagues propose novel insights to improve the performance of SLAM in complex dynamic environments in Contribution 6. Their primary approach is to impose hierarchical constraints on dynamic features through instance segmentation and multi-view constraints, preserving robust static features. The SOLOv2 instance segmentation algorithm is utilized to eliminate dynamic and potentially dynamic features, retaining reliable static features and generating a robust base matrix. The article also examines how the target semantic information obtained from the instance segmentation algorithm can be fused with a 3D semantic point cloud to create a 3D octane semantic map containing instance-level semantic information. This approach enhances the robot’s perception ability and understanding of the surrounding environments, improving the adaptability to dynamic environments.

As presented in Contribution 7, Zhang and Chen offer valuable insights into the real-time navigation and decision making in unpredictable environments of mobile robots. The authors present an enhanced version of the SAC-LSTM algorithm, incorporating a burn-in mechanism and a prioritized experience replay (PER) mechanism. The effectiveness of this algorithm is illustrated in a simulation environment. The integration of the burn-in training approach to counteract the memory degradation and the utilization of prioritized experience replay to enhance the sampling efficiency results in substantial improvements in the convergence speed and planning accuracy. This research provides a robust framework for mobile robot navigation and sets a new benchmark for future research in autonomous systems, inspiring advances and practical implementations.

The article by Jae-Bong and co-authors (Contribution 8) focuses on quadruped robots with robotic arms. The authors aim to design a quadruped mobile robot system that integrates perception, navigation, movement, and manipulation for item cleanup in households. The designed system, as presented in the article, is divided into three modules: perception, behavior control, and joint control. In the perception module, machine learning methods, specifically YOLOv7, KNN, and RANSAC, are employed to detect objects in real time and generate point clouds, enabling the estimation of optimal grasping postures. The behavior control module utilizes SLAM to construct an environment map, while the AMCL navigation package in ROS facilitates precise navigation. In the joint control module, MPC is employed to regulate the four legs, and the inverse kinematics solution controls the position of the low-degree-of-freedom manipulator. The simulation results in a virtual environment demonstrate that the authors’ system achieves an average success rate of 96% for different object classifications, indicating good stability and accuracy. However, it is important to note that the inherent advantages of the quadruped robot are not fully demonstrated due to the flat terrain of the housing environment. It is suggested that the authors consider these relevant aspects in future work.

Ahmed Neaz focuses on the development of an advanced omnidirectional mobile robot designed to enhance industrial logistics tasks in dynamic and heavy-traffic environments. Contribution 9 presents an integrated control system that combines high-level and low-level algorithms with a graphical interface for each system. The low-level motor control is achieved using an efficient microcontroller to ensure high accuracy and robustness. High-level decision making is managed by a Raspberry Pi 4 and a remote PC, utilizing multiple LiDAR sensors, IMU, and odometry data from wheel encoders. The low-level programming is implemented in LabVIEW, while the high-level software architecture uses ROS. The practical operation of the ’MotionBot’ robot demonstrates the reliability and effectiveness of the proposed techniques. Enhancements in lower-level control optimize the vibrations and increase the stability with a low computational cost. The robot showcases robustness across various environments and loads, and the fusion of data from three LiDAR sensors significantly improves the localization and positioning accuracy. The robot successfully navigates to the target points while avoiding obstacles in dynamic settings, supported by a user-friendly GUI developed in LabVIEW. Overall, this study presents a comprehensive and robust solution for autonomous navigation and mapping in industrial logistics, with significant potential for real-world applications.

Contribution 10 explores the distributed containment control of continuous-time linear multi-agent systems. The authors introduce a novel parametric dynamically compensated distributed control protocol that incorporates information from both the virtual layer and the actual neighboring agents. By adjusting the dominant poles using MLQR optimal control and Geršgorin’s circle criterion, the containment control achieves a specified convergence speed. Furthermore, the authors address the adaptability of the dynamic control protocol in the event of the failure of the virtual layer, ensuring that the convergence speed can still be guaranteed through dominant pole assignment methods. The study emphasizes the importance of dynamic performance tuning, the design of distributed control protocols for MASs with multiple leaders over fixed topology, and the significance of the convergence speed for the system’s stability and performance.

In Contribution 11, Jie Meng and co-authors consider the relationship between perception and control. They propose a novel MPC framework to enhance the tracking accuracy in the presence of localization fluctuations. The prediction horizon is time-varying, following the localization accuracy, mitigating the effects of localization fluctuations, and improving the tracking accuracy and system stability in dynamic environments. The authors also introduce a methodology for assessing the fluctuations in localization, employing fuzzy rules to estimate the variance and entropy, providing a means of quantifying the magnitude of localization fluctuations. To reduce the computational burden and ensure the feasibility of the optimization problem, a Taylor expansion is employed to linearize the kinematic model of the robot. This approach represents a novel conceptual framework compared to the traditional method of decoupling perceptual and control methods, as it effectively enhances the tracking performance of the robot in the presence of perceptual uncertainty.

A simple and easy-to-implement localization system is essential for robots. Therefore, in Contribution 12, a visual tag-based indoor localization system is proposed. It is important to construct the tags straightforwardly to minimize the algorithm’s complexity. However, this often leads to non-uniqueness issues with the tags. To address this, the authors propose a methodology for efficient tag matching based on the azimuth angle between the camera and the tag. Additionally, a method for estimating the tag’s position is devised to achieve an optimal balance between computational complexity and positioning accuracy.

Huma Mahboob introduces a novel approach to autonomous navigation in unknown environments in Contribution 13. Instead of focusing on the local optimization of individual robots, the proposal aims to optimize the overall energy consumption of a population of robots. An innovative energy- and information-aware management algorithm is proposed, enabling each robot to draw and update a map of the entire environment by receiving information broadcasted by other robots. By comparing this with their own environment perception, the robots can determine their position on the map, enabling real-time localization. By guiding the robots’ perception of their optimal paths, sharing sensory information among followers, and comparing energy consumption under information transfer and collaborative perception, the robot swarm can reach its destination with minimal energy consumption. This approach offers valuable insights for the advanced fields, with a notably low overhead in collaborative perception, real-time mapping, localization, and navigation.

The final contribution is a review by Yu Liu and co-authors, which presents the technologies used for perception by indoor autonomous mobile robots. The authors emphasize the significance of perception in mobile robotics and the need for accurate and efficient sensing capabilities to make informed decisions. The review provides a systematic literature review, covering various techniques for robot localization, including inertial navigation, GPS, navigation based on beacons or landmarks, and model matching. The article also discusses map-building techniques, with a focus on SLAM methods, including filter-based and graph-optimization-based algorithms. Additionally, the importance of LiDAR and vision cameras in SLAM, as well as the processing of SLAM optimization algorithms, is highlighted. In conclusion, the review emphasizes the importance and application of perception techniques, localization methods, and map-building algorithms for mobile robots operating in indoor environments.

## 3. Conclusions

This collection of papers on advanced sensing and control technologies for autonomous robots provides valuable insights into the current state of research and the state of the art in the field. The authors’ contributions provide distinctive perspectives and innovative solutions to address pressing challenges in robotics. Building on the preceding discourse, several potential avenues warrant further exploration to establish robust and reliable navigation capabilities in sensing and control technologies. To this end, the following recommendations are presented for consideration:

- **Multi-sensor data fusion:** The inherent limitations of a single sensor pose significant challenges in the deployment of autonomous robots in complex scenarios, such as intelligent transport systems. Consequently, the integration and analysis of data from multiple sensor sources are imperative to generate comprehensive, accurate, and reliable information. This will involve addressing data heterogeneity and uncertainties from a broad range of sensors, ensuring the integrity and trustworthiness of the fused information, and advancing the development of productive fusion algorithms to enable a rapid response when needed.
- **Perception-control coupling method:** Autonomous robots complete some specific tasks in which accurate control is required, such as safe navigation under interference, the sensor faults of aircrafts, and the palletizing tasks of industrial robots. In current robotics systems, the perception and control layers often operate as distinct components. Notably, the localization results of the robot are typically considered accurate within the control framework. However, in practical applications, external errors persist, whether associated with LiDAR-based or vision-based localization strategies. In the absence of an appropriate strategy on the part of the controller, these errors can lead to severe system instability or substantial tracking deviations. Therefore, formulating a well-structured perception-control method becomes paramount to effectively mitigate such problems.
